# Moving Towards Induced Pluripotent Stem Cell-based Therapies with Artificial Intelligence and Machine Learning

**DOI:** 10.1007/s12015-021-10302-y

**Published:** 2021-11-29

**Authors:** Claudia Coronnello, Maria Giovanna Francipane

**Affiliations:** 1 Advanced Data Analysis Group, Fondazione Ri.MED, 90133 Palermo, Italy; 2 Regenerative Medicine Group, Fondazione Ri.MED, 90133 Palermo, Italy; 3 McGowan Institute for Regenerative Medicine, University of Pittsburgh, Pittsburgh, PA 15232 USA

**Keywords:** Induced pluripotent stem cells, Regenerative medicine, Quality control, Artificial intelligence, Machine learning, Deep learning

## Abstract

The advent of induced pluripotent stem cell (iPSC) technology, which allows to transform one cell type into another, holds the promise to produce therapeutic cells and organs on demand. Realization of this objective is contingent on the ability to demonstrate quality and safety of the cellular product for its intended use. Bottlenecks and backlogs to the clinical use of iPSCs have been fully outlined and a need has emerged for safer and standardized protocols to trigger cell reprogramming and functional differentiation. Amidst great challenges, in particular associated with lengthy culture time and laborious cell characterization, a demand for faster and more accurate methods for the validation of cell identity and function at different stages of the iPSC manufacturing process has risen. Artificial intelligence-based methods are proving helpful for these complex tasks and might revolutionize the way iPSCs are managed to create surrogate cells and organs. Here, we briefly review recent progress in artificial intelligence approaches for evaluation of iPSCs and their derivatives in experimental studies.

**Graphical Abstract:**

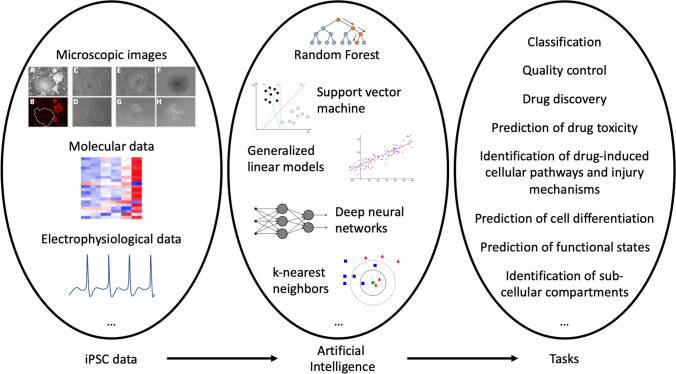

## Methods

A comprehensive literature search was conducted on May 21, 2021 using the PubMed-NCBI database. The following search terms were used: (1) induced pluripotent stem cells[MeSH:noexp] AND artificial intelligence[MeSH:noexp]; (2) induced pluripotent stem cells[MeSH:noexp] AND deep learning[MeSH:noexp]; (3) induced pluripotent stem cells[MeSH:noexp] AND machine learning[MeSH:noexp]. PubMed search returned a total of 29 results. Three non-English (Japanese) language publications, one review, and four editorial/commentary contributions were excluded from the manuscript. Further search in the Scopus database returned an additional publication, which was also included. During manuscript revision, we added seven more articles as suggested by the reviewers. Considered articles were published between 2014 and 2021.

## Introduction

Failed or failing organs, according to well-established practice, may be replaced by healthy ones obtained from a cadaveric or a live donor. Success of this approach, as significant as it is, however, is restricted by the short supply of donors of either type.

In recent years, alternative approaches for functional organ generation have emerged. Organ generation using tissue-specific stem/progenitor cells has been suggested [1], and more recently, induced pluripotent stem cells (iPSCs) have opened new avenues for regenerative treatments [2]. iPSCs hold great potential for the development of personalized therapies without the ethical issues associated with embryonic stem cell treatment and the immunological risk of rejection. This promise has spurred efforts to generate all known cell types for therapeutic purposes, which have resulted in a hundred of clinical trials (http://clinicaltrials.gov). However, major drawbacks for clinical translation are the low reprogramming and differentiation efficiency of common iPSC protocols [3], as well as the high variability in differentiation outcomes [4], and the occurrence of differentiation-defective phenotypes [5]. iPSCs, during early culture passages, have a residual epigenetic memory of the tissue from which they were derived [6], and might revert to their somatic cells of origin. Furthermore, the genomic instability associated with the reprogramming process [7], and/or small variations in the complex multistep culture system [8], can influence iPSC response to differentiation stimuli and, hence, cell fate decisions. In many cases, the progeny of iPSCs are comparable to an immature fetal stage [9–11]. Failure to provide mature and functional cells, or contamination of the cellular product with residual undifferentiated iPSCs, might be detrimental to the recipients of iPSC-based therapies.

For safe and effective autologous cell replacement, a thorough evaluation of the iPSC-derived cell product at different stages of culture is required. The current solution relies on a judgement call from well-trained cell culture experts, who often determine iPSC induction and maturation based on changes in morphology and/or lineage marker expression, tasks which are extremely effort-intensive and subjectively biased. Scalable production of therapeutic cells cannot be based on manual cell quality control. An automated method enabling high-throughput validation of cell identity and function would be desirable throughout the entire manufacturing process. The screening is multifold. It is needed: (1) in the reprogramming stage, to select those somatic cells which have been fully converted to iPSCs; (2) in the expansion stage, to exclude abnormal or unstable iPSC colonies; and (3) in the differentiation stage, to select functional mature cells for implantation.

While practical application of iPSCs in the clinic may not be forthcoming, an automated, high-throughput method would at least sustain the use of iPSC derivatives as drug screening platforms, by helping understand how drugs impact key cellular functions.

Developments in digital pathology and computational image analysis have provided advanced tools for cellular morphology description and classification [12]. Given the high-dimensionality of the data generated by computational image analysis, artificial intelligence, with the use of machine learning algorithms, has been increasingly deployed to build cell image classification methods [13]. Machine learning algorithms are able to learn from large datasets and to make predictions based on novel input. Hence, they can evaluate multiple parameters simultaneously without a priori knowledge. Several different machine learning methods have been developed in the last fifty years. Few examples are: the nearest-neighbor search developed in the 1960 s [14], support vector machines (SVM) in the 1990 s [15], and random forest (RF) in the early 2000 s [16].

In the machine learning field, deep learning has also attracted much attention. Deep learning uses a multilayered neural network that mimics human neural circuit structure [17]. Deep neural network can automatically extract features from an image, while traditional machine learning methods require human intervention. Convolutional neural network (CNN)-based deep learning methods or *convnets*, are now used for a wide range of image-related tasks. Such methods transform input images into predicted outputs after learning the proper associations from examples. Their performance largely depends on the features extracted for a given task, and it is usually measured using statistical metrics such as accuracy, precision, recall, F1 score, the receiver operator characteristic curve (ROC), and the area under the curve (AUC).

Not only applied to biological images, but also machine learning techniques have started to be exploited for the processing and the analysis of the huge amount of data or *big data*, that is being created by advancements in next generation sequencing (NGS) technologies in various areas of medicine including the iPSC field [18].

By helping evaluate both the reprogramming state and the differentiation trajectories of human iPSCs, machine learning and deep learning have the power to open up the game for greater iPSC bioprocess efficiency and yield. A review of the methods which have been adopted in research for the identification, classification and prediction of iPSCs follows.

### Machine and Deep Learning Methods for Image-based iPSC Identification and Functional Characterization

Recently, machine learning methods have been trained to predict iPSC induction and differentiation from microscopy images. Machine learning methods based on time-lapse images of the morphology and motion pattern of iPSCs were used to predict/identify iPSCs against feeder fibroblasts during the early stage of the reprogramming process [19]. After 48 h of infection, the reprogramming process was recorded using a live cell imaging system. iPSCs and feeder fibroblasts within 3 to 5 days after infection were then labeled by retrospectively tracing the time-lapse microscopic image. Eleven types of cell morphological and motion features (volume, area, sphericity, ellipsoid-prolate, ellipsoid-oblate, nucleus-cytoplasm volume ratio, displacement, speed, etc.) were calculated, and different time windows were considered for modeling and perform feature selection. Six features and best time windows were finally used to build a prediction model using the algorithm XGBoost. In another study, the quality of newly reprogrammed iPSC colonies was identified from phase-contrast images using SVM followed by the feature extraction method Scaled Invariant Feature Transformation (SIFT) [20]. In these images, feeder cells were also included. The classification task was a multiclass problem with three possible classes (good/semigood/bad) for the iPSC colonies. Importantly, such colony image classification method could be improved by applying an error-correcting output code (ECOC) framework [21]. Other authors developed a model to guide colony selection using a combination of bright-field microscopic images and CNNs [22]. Specifically, the CNN model was trained to locate unlabeled iPSC colonies and detect their boundaries. After the boundary of a colony was found, each colony was measured in terms of the area and time frame after reprogramming induction, and a growth curve was plotted. Abnormal growth conditions (overgrowth/undergrowth) were manually defined and normal colonies were used to train a Hidden Markov Model (HMM) for prediction of optimal picking time window.

Healthy quality of undifferentiated iPSCs is an essential requisite for further experimental and therapeutic approaches. Kavitha et al. developed a vector–based CNN (V-CNN) to classify healthy from unhealthy colonies, considering both colony morphological and textural features [23]. In a further study, 151 texture features, extracted quantitatively from segmented colony regions, were evaluated using several machine learning classifiers [24]. This approach could achieve a robust and reliable classification accuracy in the range of 82.5–92.7%, with low false positive and negative rates.

Not only for colony detection and classification, but also machine learning has been exploited to reveal specific iPSC cellular constituents. Indeed, Christiansen et al. designed a deep neural network capable of predicting fluorescent labels against nuclei or cell-type-specific markers from the z-stack of unlabeled transmitted-light images of fixed and live iPSCs [25]. Cellular constituents of several types of cells, including iPSCs could also be recognized in three-dimensional (3D) tissues by the CNN-based Cell Profiler 3.0 software, which supports both whole-volume and plane-wise analysis of 3D image stacks [26].

While the above described machine learning methods require to specify target morphologies, choose specific algorithms, and try different parameters depending on the imaging problem, the open source utility wndchrm, i.e. weighted neighbor distances using a compound hierarchy of algorithms representing morphology, provides an automated pipeline [27]. Wndchrm allows users to define classes by providing example images for each class; completely reprogrammed cells or partially reprogrammed cells, for example. Given that nuclear morphology changes during differentiation status, Tokunaga et al. constructed wndchrm image libraries from immunofluorescence of the promyelocytic leukemia (PML) and Cajal bodies to discriminate bona fide iPSCs from non-iPSCs [28].

Beside supporting iPSC colony identification/prediction/classification, machine learning methods might also help assess differentiation and function of iPSC-derived cells. CNNs were trained to predict whether phase-contrast images contained human iPSC-derived endothelial cells (hiPSC-ECs) based on morphology only [29]. Predictions were later validated by comparison with immunofluorescence staining for CD31, a pan-endothelial marker. Using high-throughput image-processing and SVM, Smith et al. considered instead the relationships between cytoskeletal tension, density, and micropattern geometry to predict pattern formation in early and late-stage human iPSC maturation toward both endothelial cells and pericytes [30]. Furthermore, a few studies used artificial intelligence methods to assess the quality of human iPSC-derived cardiomyocytes (hiPSC-CMs). Orita et al. trained CNNs using bright-field images of hiPSC-CMs to classify the images into normal (experimentally useable) or abnormal (experimentally unusable) [31]. Lee et al. established a screening method that combines bright-field microscopy and machine learning to detect changes in the contraction of hiPSC-CMs after exposure to three cardioactive drug compounds with distinct, dissimilar effects: E-4031 (hERG K^+^ channel inhibitor), verapamil (L-type Ca^2+^ channel blocker), and blebbistatin (myosin-II inhibitor) [32]. For the bright-field method, images were processed by an optical flow algorithm to generate vectors that represent the motion of hiPSC-CMs. The optical flow method was later combined with SVM. SVM classified the data points into normal and abnormal cardiomyocyte behavior by creating a decision boundary between the two groups. Another method to assess the quality of hiPSC-CMs consisted in optical quantification of the contractility of hiPSC-CMs using bright-field microscopic videos [33]. Contraction waves were extracted directly from time-lapse video images using Fiji image processing package in ImageJ, and were divided into normal contraction (experimentally useable) and abnormal contraction (experimentally unusable) waves using an SVM classification. In addition to contractility, Ca^2+^ transients were also exploited for functionality assessment of hiPSC-CMs. Indeed, calcium cycling has a central role in cardiac functionality by linking electrical activation and contraction. Juhola et al. first proposed an analytical algorithm to detect cycling Ca^2+^ transient peaks, quantify peak variables, and assess the abnormality of transient peaks and signals using iPSC-CMs generated from genetic cardiac disease patients [34]. However, signal abnormality was based solely on characteristics of a single peak. An improved method consisting in the identification of peak abnormality based on quantified peak characteristics, was later suggested by Hwang et al. [35]. Ca^2+^ transient data of 200 cells and 1893 peaks were collected and analyzed to train peak- and cell-level SVM models, and later validated using the leave-one-out cross-validation (LOOCV) approach. In parallel, test data of 54 cells and 454 peaks were used to implement the SVM classifier to predict cell abnormality. This machine learning classification method obtained higher sensitivity and accuracy with respect to the previous analytical algorithm, and also allowed separating different genetic cardiac diseases from each other and from controls [36, 37]. The genetic cardiac diseases included: catecholaminergic polymorphic ventricular tachycardia (CPVT), long QT syndrome 1 (LQT1), hypertrophic cardiomyopathy (HCM), dilated cardiomyopathy (DCM), and long QT Syndrome 2 (LQT2). The improved method could also predict the type of mutation based on Ca^2+^ transient signals only [38]. Finally, machine learning was exploited to study drug responses of hiPSC-CMs. Heylman et al. used machine learning to classify the electrophysiological effects of chronotropic drugs on hiPSC-CMs based on alteration of membrane depolarization waveforms [39], while Juhola et al. used machine learning to detect drugs affecting calcium cycling properties of CPVT iPSC-CMs [40].

Besides iPSC-CMs, the iPSC-derived retinal pigment epithelium (iPSC-RPE) was also analyzed using artificial intelligence-based methods. Deep neural networks and traditional machine algorithms were used to predict iPSC-RPE function from quantitative bright-field absorbance microscopy (QBAM) images [41]. To demonstrate the effectiveness of the imaging and analysis method, a proof-of-principle study was carried out on iPSC-RPE from the following donor types: healthy, oculocutaneous albinism disorder (OCA), and age-related macular degeneration (AMD) donors. QBAM was first used to assess iPSC-RPE for transepithelial resistance (TER) and polarized vascular endothelial growth factor (VEGF) secretion, where TER is a measure of RPE maturity that increases as tight junctions form between neighboring cells, and polarized VEGF secretion is a measure of RPE function. Single-cell analysis began with a deep neural network that identified cell borders in QBAM images. Next, visual features of individual cells were extracted from QBAM images using the web image processing pipeline (WIPP). The extracted visual features were then used to train five different traditional machine learning methods (multilayer perceptron [MLP]; linear SVM; RF; partial least squares regression [PLSR]; and ridge regression [RR]) to predict a variety of tissue characteristics, including cell function, donor identity, and developmental outliers. The iPSCs-RPEs from healthy donors were imaged as they matured throughout the long culture, thus providing a comprehensive/continuous assessment, while iPSCs-RPEs from AMD and OCA donors were imaged at a terminal time point once they had reached maturity. The latter approach allowed to predict function, identity and developmental outliers just prior to implantation. Similarly, Ye et al. developed a machine learning-based prediction model to predict failure RPE products [42]. As F-actin plays an important role in the maintenance of the epithelial architecture, authors analyzed how F-actin was distributed in RPE sheets and from this data predicted TER values. Cellular morphological analyses were performed using the ImageJ plugin Cell Magic Wand. Importantly, the TER discrimination model could also predict failure samples from non-labeled images.

Machine learning approaches also proved successful in image-based analysis of cellular pathways and injury mechanisms, as demonstrated by Kandasamy et al., who combined an *in vitro* model of human iPSC-derived renal proximal tubular cells (iPSC-HPTCs) with the automated classifier RF to predict drug-induced proximal tubular toxicity in humans [43]. The nephrotoxicity prediction performance of iPSC-HPTCs was determined by evaluating their responses to 30 compounds. Given that compounds that are toxic to renal proximal tubular cells increase interleukin-6 (IL-6) and/or interleukin‐8 (IL-8) expression, nephrotoxicity was predicted by exploiting changes in the levels of these cytokines, as determined by qPCR. Not only drug-induced toxicity could be predicted, but also underlying injury mechanisms and compound-induced cellular pathways could be detected with automated imaging of γH2AX generation, 4-hydroxynonenal (4-HNE) production, and nuclear-cytoplasmic translocation of the nuclear factor (NF)-κB p65 subunit.

Thus, the power of machine learning can be leveraged in image-based characterization of iPSCs and iPSC derivatives, and support future application of iPSCs in regenerative medicine and drug discovery.

### Machine and Deep Learning Methods for Genomic-based iPSC Identification and Functional Characterization

Machine learning has been applied not only to image processing, but also to gene expression profiles. Danter et al. developed an unsupervised deep machine learning technology called DeepNEU to simulate artificial iPSC systems using a defined set of reprogramming transcription factors [44]. By employing a fully-connected recurrent neural network architecture with one processing layer for each input variable, the DeepNEU platform enabled authors to gain a better understanding of gene and pathway regulation in pluripotent and reprogrammed somatic cells, and therefore, key information about which genes/molecules are indispensable for iPSC generation and maintenance.

In addition, machine learning techniques are being increasingly exploited to extract biologically relevant transcriptomic and epigenetic signatures from NGS data. Bardy et al. built an extremely randomized trees (ERT) classifier with the transcriptome of 56 single cells and trained it with electrophysiological data to classify the functional states of human iPSC-derived neurons [45]. Wu et al., used NGS and machine learning to screen a library of 6107 synthetic promoters with enhanced cell-state specificity (SPECS) [46]. Through this approach, they identified multiple SPECS that exhibit distinct spatio-temporal activity during iPSC differentiation.

Another example of network-based screening that leverages iPSC and machine-learning technologies has been very recently given by Theodoris et al. in the context of aortic valve (AV) disease, which is caused by heterozygous loss-of-function NOTCH1 (N1) mutations [47]. ECs are drivers of AV disease and therapeutic targets. To map the gene network disrupted by N1 haploinsufficiency and to identify small molecules that could correct the network back to a normal state, the authors designed a targeted RNA-seq strategy assaying expression of 119 signature genes in N1+/– iPSC-ECs or gene-corrected isogenic cells exposed to either dimethyl sulfoxide (DMSO) or one of a panel of 1595 small molecules. Next, the authors trained a K-nearest neighbors (k-NN) algorithm to classify the gene expression network by targeted RNA-seq as WT or N1+/– based on isogenic ECs of each genotype exposed to DMSO. The k-NN algorithm classified ECs as either WT or N1+/– with 99.3% accuracy by LOOCV. Authors next applied the trained k-NN algorithm and hierarchical clustering to N1+/– ECs exposed to a library of 1595 small molecules to identify those molecules that could shift gene expression networks such that treated N1-haploinsufficient ECs could cluster with WT ECs. Through this investigation, they identified eight compounds that could correct gene expression networks such that one or more replicates of treated N1+/– ECs were classified as WT by the k-NN algorithm in validation trials. Of these, XCT790, an inverse agonist of estrogen-related receptor alpha (ERRα), had the strongest restorative effect.

Over the last several years, machine learning has also been applied to CRISPR/Cas9 system, the third-generation genome editing technology. An example is provided by Liu et al. [48], who developed a CRISPR interference (CRISPRi) platform targeting 16,401 long non-coding RNA (lncRNA) loci in diverse cell lines including human iPSCs, and conducted screens for IncRNA genes that could modify cell growth. Large-scale screening identified 499 lncRNA loci required for robust cell growth. Growth modifier lncRNA function was found to be highly cell type-specific. Interestingly, a larger fraction of lncRNAs hits were observed in the iPSC screen, suggesting that iPSCs are either more susceptible to growth perturbations or are differentiating to other cell types with lower growth rates. Taking advantage of the large dataset, authors finally constructed generalized linear models to assess which genomic properties could be predictive of lncRNA function and found an association of lncRNA function with higher order chromatin structure.

Overall, this evidence demonstrates how the extremely cumbersome manufacturing process for iPSC-derived functional cells is forcing researchers to leverage functional genomics and cutting-edge artificial intelligence algorithms to drill into the biology of iPSCs.

## Conclusions

Since its beginning fifteen years ago [49], iPSC technology has evolved rapidly. Currently, different studies are exploring its potential application in regenerative medicine. However, there is still no solid strategy ensuring the exclusion of contaminants such as residual undifferentiated iPSCs from differentiated cell products. Candidate marker genes for detecting undifferentiated iPSCs have been recently selected from single cell RNA sequence data [50]. Yet, this strategy has limitations with regard to the amount of product that can be validated in each assay.

In our experience, maintaining normal (useable) iPSC colonies in vitro is very challenging. First, iPSC colonies must be manually picked and re-plated from the primary reprogrammed cultures. Live immunostaining for Tra-1-60, a surface marker of pluripotent cells, can help identify true iPSC colonies. In our graphical abstract, A and B microscopic images show Tra-1-60 immunofluorescence staining and phase contrast respectively of a primary reprogrammed culture. Absence of expression of Tra-1-60 in a colony (dashed line) indicates that it is not fully reprogrammed. In the early passages, iPSCs often undergo spontaneous differentiation. Normal (usable) from abnormal (unusable) colonies can be easily distinguished based on morphology. C and D microscopic images show normal iPSC colonies. These colonies appear flat and compact, and show distinct borders. E-H microscopic images represent abnormal iPSC colonies. These colonies show irregular morphologies and/or signs of (de)differentiation, which can be appreciated at the colony center (E and F) or at the colony edges (G and H). A glandular-like phenotype can be observed in image F, which might be indicative of spontaneous endoderm differentiation. Image G shows the presence of contaminating unreprogrammed cells in the well, while image H shows fibroblast-like spindle-shape cells at the borders of a colony. When such abnormal colonies appear in the culture, it is important to remove them promptly.

Effective differentiation is highly dependent on iPSC quality. As such, several critical decisions must be taken when cultivating iPSCs, including but not limited to, when it is the right time to passage the colonies, which is the proper cell aggregate size during passaging, and what is the best colony density for maintaining healthy undifferentiated iPSCs in vitro. These properties might be specific to each cell line and must be therefore experimentally determined. Accumulating evidence suggests that artificial intelligence, which applies machine learning, deep learning and other techniques to solve complex problems, might help answer these questions. Several machine learning approaches have already been developed and their significance in classifying iPSCs and their derivatives has been confirmed. In this manuscript, we have provided an overview of machine learning-based state-of-the-art methods in such a rapidly evolving field, which we have summarized in Table 1.

**Table 1:** Machine learning applications in the iPSC field. For each given example, information is provided about input data, expected output, as well as the feature extraction and selection techniques, and the prediction tools used

Task	Input	Output	Feature extraction	Feature selection		Prediction			Reference
Identification / Prediction of iPSCs	Time-lapse microscopic images; 11 types of morphology and motion features	iPSCs / feeder fibroblasts	Imaris software	Recursive feature elimination		XGBoost			[19]
Classification of iPSC colonies	Phase-contrast images	Bad / semigood / good quality	SIFT	NLOO		SVM and *k*-NN			[20]
Classification of iPSC colonies	Phase-contrast images	Bad / semigood / good quality	SIFT	NLOO		*k*-NN, LDA, QDA, NB, CART, with ECOC			[21]
Identification of iPSC colonies	Time-lapse based-bright-field microscopic images	iPSC colony boundaries	DCNN						[22]
Prediction of optimal iPSC colony selection time	Time-lapse based-bright-field microscopic images	Phase of growth curve	n.a.		Manual selection			HMM	[22]
Classification of iPSC colonies	Phase-contrast images	Healthy / unhealthy colonies	Morphology and texture		V-CNN				[23]
Classification of iPSC colonies	Phase-contrast images	Healthy / unhealthy colonies	151 texture features	Stepwise regression		SVM, RF, MLP, DT, Adaboost			[24]
Prediction of fluorescent labels against specific cellular constituents in unlabeled images	Transmitted-light z stacks of cells fluorescently labeled (Hoechst, DAPI, CellMask, Propidium iodide, TuJ1, Islet1, MAP2, pan-axonal neurofilaments)	Location and texture of cell nuclei, cell health, the type of cell in a mixture, and the type of subcellular structure	DNN						[25]
Identification of iPSC colonies and sub-cellular compartments	3D image stacks of DNA stains	Nuclei segmentation (background / nucleus interior / nuclear boundary)	CNN (Unet implemented in CellProfiler software)						[26]
Identification of iPSC colonies	Phase contrast images and immunofluorescence images of nuclear structures	Bona fide iPSC (completely reprogrammed) / non-iPSC (incompletely reprogrammed)	wndchr						[28]
Prediction of iPSC differentiation towards endothelial cells	Phase-contrast images (cell morphology)	Differentiation towards endothelial cells	CNN (LeNet, AlexNet)						[29]
Prediction of pattern formation in early and late-stage iPSC maturation toward vascular lineages	Cytoskeletal tension, density, and micropattern geometry tuned through interference of the RhoA/ROCK pathway	Differentiation towards endothelial cells or pericytes	n.a.	n.a.			SVM		[30]
Assessment of the quality of iPSC-CMs	Bright-field images	Normal / abnormal differentiation	CNN						[31]
Assessment of the quality of iPSC-CMs	Time-lapse brightfield and fluorescence microscopic images	Normal / abnormal contractions	Optical flow algorithm	n.a.		SVM			[32]
Assessment of the quality of iPSC-CMs	Bright-field microscopic videos	Normal / abnormal contractions	Fiji - ImageJ	UMAP		SVM			[33]
Detection of disease-specific iPSC-CMs (CPVT)	Ca2+ transient data	Normal / abnormal Ca2+ transients	Peak attributes	n.a.		*k*-NN, LDA, QDA, DAM, NB, NBK, SVM			[34]
Assessment of the quality of iPSC-CMs	Ca2+ transient data	Normal / abnormal Ca2+ transients	MetaXPress	n.a.		SVM			[35]
Detection of disease-specific iPSC-CMs (LQT1 / HCM / CPVT)	Ca2+ transient data	Normal / abnormal Ca2+ transients	Peak attributes	n.a.		k-NN, RF, LS-SVM			[36]
Classification of disease-specific iPSC-CMs (LQT1 / LQT2 / HCMM / HCMT / CPVT / DCM)	Ca2+ transient data	Normal / abnormal Ca2+ transients	Peak attributes	n.a.		*k*-NN, LDA, QDA, CART, NB, LS-SVM, ECOC			[37]
Classification of disease-specific iPSC-CMs (HCMM / HCMT LQT1 / LQT2)	Ca2+ transient data	Normal / abnormal Ca2+ transients	Peak attributes	n.a.		*k*-NN, LDA, QDA, NB, CART, LS-SVM			[38]
Assessment and classification of chronotropic drug effects on iPSC-CMs	High temporal resolution 2-photon microscopy	Drug exposure based on membrane depolarization waveforms	TreeBagger (RF with bootstrap aggregation) - Matlab						[39]
Detection of drugs affecting Ca2+ cycling properties of CPVT iPSC-CMs	Ca2+ transient data	Drug effects	n.a.	One-way variance analysis					[36]
Prediction of iPSC-RPE function	QBAM images	Function (TER and VEGF-ratio), identity and developmental outliers	WIPP	MLP; linear SVM; RF; PLSR; RR					[41]
Prediction of iPSC-RPE function	F-actin-labeled microscopic images	Failure samples based on predicted TER values	Cell Magic Wand-ImageJ						[42]
Prediction of drug-induced nephrotoxicity in iPSC-HPTCs	IL-6 and IL-8 qPCR data	Toxic / non-toxic compound	n.a.	n.a.		RF			[43]
Identification of drug-induced cellular pathways and injury mechanisms in iPSC-HPTCs	Automated imaging of γH2AX generation, 4-HNE production, nuclear-cytoplasmic translocation of the NF-κB p65 subunit	Compounds inducing DNA double strand breaks, reactive oxygen species and inflammation	n.a.	n.a.		RF			[43]
Simulation of iPSC systems using a defined set of genes/proteins	3589 genes / proteins involved in hESC pathways and 27,566 gene / protein regulatory relationships important in hESCs	Expression or repression of genes and proteins in iPSCs	Fully-connected recurrent neural network						[44]
Prediction of the functional states of human iPSC-derived neurons	PatchSeq data	Less functional / more functional neurons based on predicted action potentials	n.a.	PCA		ERT classifier			[45]
Prediction of promoter activity during iPSC differentiation	NGS and computational data of FACSorted mKate2 positive (synthetic promoter-expressing) cells	mKate2 fluorescence intensity	n.a.	GLMNET					[46]
Identification of small molecules able to revert the gene expression profiles of AV iPSC-ECs back to a normal state	RNA-seq expression profile of 119 genes in N1+/– iPSC-derived ECs or gene-corrected isogenic cells exposed to either DMSO or a panel of small molecules	WT or dysregulated gene network after drug exposure	n.a.	PCA		*k*-NN			[47]
Identification of cell growth-modifying lncRNAs	Large-scale screening data of lncRNA genes	Functional / non-functional IncRNA; genomic properties associated to IncRNA function	n.a.	GLM					[48]

**Abbreviations**: AV iPSC-ECs: Aortic valve induced pluripotent stem cell-derived endothelial cells; CNN: Convolutional neural network; CPVT iPSCs-CMs: Catecholaminergic polymorphic ventricular tachycardia induced pluripotent stem cell-derived cardiomyocytes; DCM: dilated cardiomyopathy; DCNN: Deep Convolution neural network; DMSO: Dimethyl sulfoxide; DNN: Deep neural network; DT: Decision tree classifier; ECOC: Error-Correcting Output Code; ERT: Extremely randomized trees; FACS: Fluorescence-activated cell sorting; GLM: Generalized Linear Model; GLMNET: Generalized Linear Model with elastic net regularization; hESC: Human embryonic stem cells; HCM: Hyperthophic Cardiomyopathy; HCMM: Hyperthophic Cardiomyopathy carrying MYBPC3 mutation; HCMT: Hyperthophic Cardiomyopathy carrying TPM1 mutation; HMM: Hidden Markov Model; 4-HNE: 4-hydroxynonenal; IL-6: Interleukin 6; IL-8: Interleukin 8; IncRNA: Long non-coding RNA; iPSC-CMs: induced pluripotent stem cell-derived cardiomyocytes; iPSC-HPTC: induced pluripotent stem cell-derived human primary renal proximal tubular cells; iPSC-RPE: induced pluripotent stem cell derived retinal pigment epithelium; LDA: Linear Discriminant Analysis; LQT: Long QT syndrome; LQT1: Long QT syndrome carrying KCNQ1 mutation; LQT2: Long QT syndrome carrying KCNH2 mutation; K-NN: K-nearest neighbors; MAP2: Microtubule-associated protein 2; MLP: Multilayered Percepton; N1: NOTCH1; NB: Naïve Bayes; NBK: Naïve Bayes with kernel; NF-κB: Nuclear factor-κB; NLOO: Nested leave-one-out; PCA: Principal component analysis; PLSR: Partial least squares regression; QBAM: Quantitative bright-field absorbance microscopy; QDA: Quadratic Discriminant Analysis; RF: Random forest; RR: Ridge regression; SIFT: Scaled Invariant Feature Transformation; SVM: Support Vector Machine; TER: Transepithelial resistance; VEGF: Vascular endothelial growth factor; WIPP: Web image processing pipeline; WT: Wild type

Compared to humans, artificial intelligence-based methods bring enormous improvements in terms of accuracy, speed of data analysis, and costs. As such, they have the potential to lay the groundwork for an iPSC manufacturing revolution, by providing cost-effective, rapid and robust methods for efficient screening of large numbers of iPSC lines and their derivatives. This is crucial for the derivation of cells suitable for clinical applications. Furthermore, artificial intelligence-based methods can be applied in the context of iPSC-based drug discovery to assist with prediction of efficacy, toxicity and pharmacokinetics of drugs.

Not only modern artificial intelligence methods such as deep learning might provide an aid to human operator, but also, they might one day support or even replace decision making. However, much groundwork is still needed before these methods can be applied into the clinical realm. A major limitation is the need for large amounts of hand-crafted, structured training data, and this data must be good enough to yield meaningful results.

## Data Availability

Not applicable.
